# Correspondence

**Published:** 1888-07

**Authors:** W. Buzzell

**Affiliations:** Port Clinton, O.


### CORRESPONDENCE.

PORT CLINTON, O., April 5, 1888.

EDITOR OF REGISTER,

*Dear Sir:*—In compliance with your request I send you the following incomplete report.

*Case* 1. A lady between thirty-five and forty years of age came to me to have a left upper lateral incisor extracted. I found a putrescent pulp, considerable swelling of the adjacent parts, and much tenderness on percussion. Being unable to gain her consent to treat and fill, I extracted it. Then I cleaned out and disinfected the pulp canal and filled it with gutta-percha, bathed the root and socket with tepid carbolic acid solution and replaced the tooth. She reported slight pain and some soreness for a day or two, but that soon subsided, and she has now been wearing the tooth upwards of four years.

*Case* 2. A similar case in the person of a boy about sixteen, who refused to allow me to treat a tooth in the usual way because of the expense. I induced him to become the subject of an experiment by promising not to charge him any thing. (It is remarkable what a difference that makes!) I extracted, cleaned, filled, and replanted as I did the first, and the tooth was still in use a year or two afterward. I have lost track of it now.

*Case* 3. In attempting to extract a broken upper first-molar from the mouth of a girl fourteen or fifteen, while under the influence of nitrous oxide, on account of the struggles of the patient, I accidentally took out the second bicuspid. After about an hour I gained her consent to let me replant it. I took out the pulp (fearing failure to unite and consequent decomposition) filled with gutta-percha and replanted. This was nearly four years ago and it is still doing well.

*Case* 4. Another case of accidental extraction of a lower second bicuspid. This I replanted without removing the pulp. Patient called a few weeks after and I found that union had taken place. Have not heard from it since.

*Case* 5. A stout, young, healthy farmer came to me with a tooth to be extracted. I found a putrescent pulp and consequent pericementitis. I extracted, treated as before, and replaced it. It pained him that evening and he removed it. First failure.

*Case* 6. A healthy miss of about seventeen, had a lower first-molar extracted. I filled as before and replanted. Union soon appeared complete, but she came to me about a year afterward to have it removed again. I found that the alveolus had been completely absorbed from about and between the roots and on removing it found the roots deeply pitted by the absorptive process. Second failure.

*Case* 7. A man in evident ill health, ill nourished and anæmic, came with a similar case. I extracted, treated as before, and replanted. Saw him a few days after and found that he had borne it for a day or two and then removed it.

*Case* 8. The last case that I have replanted was in August, 1886. The patient was a young man of about twenty, who had a lower bicuspid with a putrescent pulp. I treated as before and replanted. He reported considerable soreness for a day or two, but this subsided and the tooth is still doing service.

I might mention as an item of psychological interest that "exemption from taxation" was the inducement in each case to submit to the experiment.

*Case* 9. A lady came to me complaining of pain in two of her incisors, which for pecuniary reasons she had had filled with amalgam. I was unable to discover any other cause for the pain and suggested that it might be due to the presence of the amalgam. Inquiry elicited the information that the teeth had pained her more or less ever since the amalgam had been inserted. I obtained her consent to remove the filling, and refilled with cement. Some time afterward she told me that the teeth had ceased to trouble her. This was four years ago, and I have not seen the patient since. This is the only case I ever met where pain seemed referable to the presence of the amalgam. I use amalgam for very much of my filling except in the anterior teeth. I am compelled to use amalgam or the forceps—in many cases.

One more interesting case occurs to me. A young German, just from the Father-land, came with all symptoms of putrescent pulp and incipient abscess of the right upper cuspid. Being unable to obtain his consent to treat conservatively, I extracted it. I should have mentioned that the tooth was to all appearance sound. There was absolutely no caries. Wishing to discover the cause of the trouble, I split the tooth. I found a putrid pulp, and in the upper part of the pulp chamber a spindle-shaped, detached mass of secondary dentine.

A few days ago another case apparently similar, came to me for treatment, but I cannot report fully, as I have not yet dismissed it. To relieve the pain I drilled through the neck of the tooth into the pulp chamber, which gave him some relief immediately.

I recall no other cases worthy of special mention. I may learn more perhaps of the history of the "amalgam case." The patient lives in Lafayette, Indiana, at present, I think.

Very Respectfully,

W. Buzzell.

---

The Odontological Society, of Cincinnati, met last month at the residence of Dr. C. M. Wright. The Dr. read a paper on "Medical Ethics" reviewing the resolution passed by the American Medical Association at Chicago last year and its effects upon the status of dentistry. The intellectual treat was followed by exercises in gastronomics at the generous board of the hearty host.

